# Chemical components of *Dendrobium crepidatum* and their neurite outgrowth enhancing activities

**DOI:** 10.1007/s13659-012-0103-3

**Published:** 2013-03-07

**Authors:** Cheng-Bo Li, Cong Wang, Wei-Wei Fan, Fa-Wu Dong, Feng-Qing Xu, Qin-Li Wan, Huai-Rong Luo, Yu-Qing Liu, Jiang-Miao Hu, Jun Zhou

**Affiliations:** 1103 State Key Laboratory of Phytochemistry and Plant Resources in West China, Kunming Institute of Botany, Chinese Academy of Sciences, Kunming, 650201 China; 2103 University of Chinese Academy of Sciences, Beijing, 100049 China

**Keywords:** Orchidaceae, *Dendrobium crepidatum*, bibenzyl, neurite outgrowth enhancing activity

## Abstract

15 compounds, including two new ones crepidatuols A (**1**) and B (**2**) were isolated from the stems of *Dendrobium crepidatum*. The planar structures of these compounds were elucidated by spectroscopic methods (NMR, MS, UV, and IR) and comparison with those from literatures. 10 compounds were send for enhancing activities on nerve growth factor (NGF) medicated neurite outgrowth in PC12 cells and the results indicated that crepidatuol A (**1**), confusarin and 3-(2-acetoxy-5-methoxy)-phenylpropanol showed enhancing activities at the concentration of 10.0 µM.

**Electronic Supplementary Material:**

Supplementary material is available for this article at 10.1007/s13659-012-0103-3 and is accessible for authorized users.

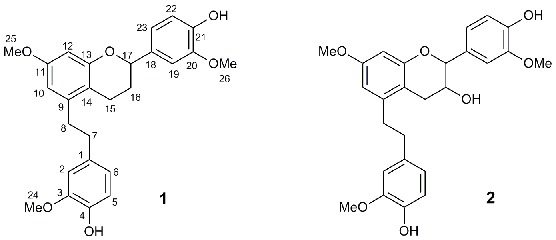

## Introduction

The stems of several *Dendrobium* species (Orchidaceae) are used as "Shi-Hu" in traditional Chinese medicine for a long time for the purpose of being beneficial to the stomach and promoting the production of body fluid, nourishing yin and clearing heat.[1,2] Inspired by this theory, "Shi-Hu" is mainly used for thrombotic diseases and health care especially for those older. *D. crepidatum* belongs to Sect. *Dendrobium* and the stems of this species used as a biological source of "Shi-Hu" and its flowers are very beautiful and get the name "Rosa Shi-Hu" in Chinese.[3] Alkaloidal part of this species has been detected by Elander etc. and five octahydroindolizine alkaloids have been isolated and elucidated.[4] To continue our research on "Shi-Hu"[5] and provide chemical evidence for this traditional Chinese medicine, *D. crepidatum* was selected as our plant material and 15 compounds, including two new compounds, crepidatuols A (**1**) and B (**2**) (Figure 1) were isolated and elucidated herein, furthermore, 10 compounds were sent to assay their neurite outgrowth enhancing activities and the results indicated that three of them showed neuroprotective effects.

## Results and Discussion

Compound **1** was obtained as yellow oil. Its molecular formula was established as $C_{26}H_{28}O_6$ by the HREIMS showing a molecular ion peak at *m/z* 436.1882 (calcd. for $C_{26}H_{28}O_6$ 436.1886), indicating thirteen degrees of unsaturation. The UV spectra at $\lambda_{max}$ (log $\varepsilon$): 282.5 (3.9) nm indicated an extended conjugated system. The $^1$H and $^{13}$C NMR spectra data of **1** (Table 1) showed the existence of three methoxy groups, four aliphatic methylenes, one aliphatic methine and three aromatic rings. Two methylene carbons at $\delta_C$ 36.2 (C-7) and 35.0 (C-8) correlated with four benzylic protons at $\delta_H$ 2.82 (4H, m, H-7 and H-8) in the HSQC spectrum of **1** indicated a bibenzyl nucleus,[6] which was confirmed by the correlations [H-7, H-8/C-1, C-2, C-6, C-9, C-10, C-14] in the HMBC spectrum (Figure 2). Three aromatic hydrogen at $\delta_H$ 6.63 (1H, d, 1.8), 6.85 (1H, d, 8.1) and 6.71 (1H, dd, 8.1, 1.8) indicated a 1,3,4-trisubstituted phenyl, moreover, two others at $\delta_H$ 6.42 (1H, d, 2.5) and 6.39 (1H, d, 2.5) showed another phenyl with two meta-coupling hydrogen for the bibenzyl nucleus. The correlations [H-16/H-15, H-17] in the COSY spectrum indicated a structural moiety of -$CH_2$-$CH_2$-CH- (C-15, C-16, C-17), furthermore, the correlations [H-15/C-14 and C-13, H-

**Figure 1.** Structures of compounds **1** and **2**

*To whom correspondence should be addressed. E-mail: hujiangmiao@mail.kib.ac.cn

**Table 1.** $^1$**H and** $^{13}$**C NMR data (500/125 MHz, δ in ppm, J in Hz) for compounds 1 and 2 in CDCl$_3$**

| | 1 | | 2 | |
|---|---|---|---|---|
| no. | $\delta_H$ | $\delta_C$ | $\delta_H$ | $\delta_C$ |
| 1 | | 133.6, s | | 133.4, s |
| 2 | 6.63 (1H, d, 1.8) | 111.1, d | 6.64 (1H, d, 1.8) | 110.9, d |
| 3 | | 146.6, s | | 146.2, s |
| 4 | | 143.8, s | | 143.8, s |
| 5 | 6.85 (1H, d, 8.1) | 114.2, d | 6.84 (1H, d, 7.9) | 114.2, d |
| 6 | 6.71 (1H, dd, 8.1, 1.8) | 120.9, d | 6.70 (1H, dd, 7.9, 1.8) | 120.9, d |
| 7 | 2.82 (2H, m) | 36.2, t | 2.81 (2H, m) | 36.2, t |
| 8 | 2.82 (2H, m) | 35.0, t | 2.81 (2H, m) | 35.1, t |
| 9 | | 141.7, s | | 142.2, s |
| 10 | 6.42 (1H, d, 2.5) | 108.2, d | 6.45 (1H, d, 2.1) | 108.9, d |
| 11 | | 158.6, s | | 158.9, s |
| 12 | 6.39 (1H, d, 2.5) | 99.5, d | 6.39 (1H, d, 2.1) | 99.1, d |
| 13 | | 156.1, s | | 155.1, s |
| 14 | | 112.3, s | | 110.8, s |
| 15 | 2.69 (2H, m) | 22.1, t | 2.63 (1H, dd, 15.6, 9.1, Ha); 3.03 (1H, dd, 15.6, 5.8, Hb) | 30.3, t |
| 16 | 2.03 (1H, m, Ha); 2.19 (1H, m, Hb) | 30.1, t | 4.02 (1H, ddd, 9.1, 8.7, 5.8) | 68.6, d |
| 17 | 4.89 (1H, d, 10.5, 1.8) | 77.5, d | 4.59 (1H, d, 8.7) | 81.6, d |
| 18 | | 133.6, s | | 129.4, s |
| 19 | 6.97 (1H, s) | 108.6, d | 6.97 (1H, d, overlapped) | 109.3, d |
| 20 | | 146.2, s | | 146.9, s |
| 21 | | 145.4, s | | 146.1, s |
| 22 | 6.93 (1H, overlapped) | 114.2, d | 6.97 (1H, d, overlapped) | 114.5, d |
| 23 | 6.92 (1H, overlapped) | 119.2, d | 6.97 (1H, d, overlapped) | 120.6, d |
| 24 | 3.92 (3H, s) | 55.9, q | 3.87 (3H, s) | 55.8, q |
| 25 | 3.76 (3H, s) | 55.2, q | 3.75 (3H, s) | 55.2, q |
| 26 | 3.86 (3H, s) | 55.8, q | 3.92 (3H, s) | 55.9, q |

16/C-14 and C-18, H-17/C-18, C-19 and C-23] in the HMBC spectrum indicated that the partial structure and the related aromatic ring were linked with the bibenzyl nucleus as trigonopol B[5a] except hydride reduction at C-16. Three methoxy groups could be observed that $\delta_H$ 3.92 (3H, s) belongs to $\delta_C$ 55.9, $\delta_H$ 3.76 (3H, s) belongs to $\delta_C$ 55.2, $\delta_H$ 3.86 (3H, s) belongs to $\delta_C$ 55.8 in the HSQC spectrum respectively, furthermore, cross peaks of $\delta_H$ 3.92 (3H, s) with $\delta_C$ 146.6, $\delta_H$ 3.86 (3H, s) with $\delta_C$ 146.2, $\delta_H$ 3.76 (3H, s) with $\delta_C$ 158.6 in the HMBC spectrum determined the position of three methoxy groups as shown in Figure 1. Thus, compound **1** was identified as 4-(5-(4-hydroxy-3-methoxyphenethyl)-7-methoxychroman-2-yl)-2-methoxyphenol, named as crepidatuol A.

Compound **2** was obtained as yellow oil, its molecular formula was determined as $C_{26}H_{28}O_7$ by the HRESIMS (*m/z* 452.1832 $[M]^+$, calcd. for 452.1835), with thirteen degrees of unsaturation. The $^{13}$C NMR and DEPT spectrum exhibited 26 carbon signals, including three methoxy, three aliphatic methylenes, two aliphatic methines and three aromatic rings. The $^1$H and $^{13}$C NMR data of **2** (Table 1) were similar to those of trigonopol B[5a] as compound **1**, indicating a similar bibenzyl core for compound **2**. The *threo*-isomer (6–8 Hz) is larger than in the *erythro*-isomer (2–4 Hz)[5e], from the chemical shifts and the coupling constants ($J$ = 8.7) of compound **2** and trigonopol B ($J$ = 6.4), it could be predicated that relative stereochemistry of C-16 and C-17 were similar in two compounds. One more methoxy in **2** than trigonopol B and correlations [H-24/C-3, H-25/C-11, H-26/C-20] in the HMBC spectrum (Figure 2) indicated that compound **2** was 5-(4-hydroxy-3-methoxyphenethyl)-2-(4-hydroxy-3-methoxyphenyl)-7-methoxychroman-3-ol as shown in figure 1, named as crepidatuol B.

The 13 known compounds were elucidated as erianin,[7] crepidatin,[8] moscatilin,[8] hircinol,[9] confusarin,[10] syringaldazine,[11] 3-(2-acetoxy-5-methoxy)phenylpropanol,[11] kaempferol,[12] 4,6-dihydroxyisobenzofuran-1(3*H*)-one,[13] ergostan-7,9,22-trien-3,5-diol,[14] ergosterol,[15] β-sitosterol and daucosterol by comparing with those from literature and TLC means.

**Figure 2.** Selected $^1$H-$^1$H COSY and HMBC correlations of compounds **1** and **2**

For the purpose of evaluating the effect of neuroprotective effects of this species, the isolated 10 compounds including crepidatuol A (**1**), erianin, crepidatin, moscatilin, confusarin, 3-(2-acetoxy-5-methoxy)-phenylpropanol, kaempferol, 4,6-dihydroxyisobenzofuran-1(3*H*)-one, ergostan-7,9,22-trien-3,5-diol and ergosterol were evaluated for their enhancing activity on NGF-induced (nerve growth factor, R&D Systems Inc., 97%) neurite outgrowth in PC12 cells.[16] The results indicated that the proportion of the NGF-induced (10 ng/mL) neurite-bearing cells were enhanced by crepidatuol A (**1**, 8.5%), confusarin (7.1%) and 3-(2-acetoxy-5-methoxy)-phenylpropanol (6.8%) at the concentration of 10 μM (Table 2). Others showed no activity on 10 ng/mL NGF-induced neurite outgrowth in PC12 cells at 10.0 μM.

## Experimental Section

**General Experimental Procedures.** Optical rotations were

**Table 2. The percentages of the neurite-bearing cells after 72 h incubation with compounds crepidatuol A, confusarin and 3-(2-acetoxy-5-methoxy)-phenylpropanol**

| group | drug | results (%) |
|---|---|---|
| 1 | blank | – |
| 2 | Negative control | 4.7 |
| 3 | Positive control | 11.9 |
| 4 | crepidatuol A | 8.5 |
| 5 | confusarin | 7.1 |
| 6 | 3-(2-acetoxy-5-methoxy)-phenylpropanol | 6.8 |

50 ng/mL of NGF was used as positive control and 10 ng/mL of NGF was used as negative control, concentration of three compounds was at 10 $\mu$M.

measured using a JASCODIP-370 polarimeter. UV spectra were obtained with a Hitachi UV-3210. IR spectra were recorded on a Bio-Rad BRUKER TENSOR27 IR spectrophotometer as KBr disks. NMR spectra were run on Bruker AVANCE 400 NMR spectrometer (400 MHz for $^1$H NMR, 100 MHz for $^{13}$C NMR) and DRX-500 NMR spectrometer (500 MHz for $^1$H NMR, 125 MHz for $^{13}$C NMR) with TMS as internal standard. Mass spectra were performed on a AutoSpec Premier P776 mass spectrometer. Column chromatography was carried out on silica gel H60 (Qingdao Haiyang Chemical Group Corp., Qingdao, China), Sephadex LH-20 (Amersham Biosciences), and ODS (40–63 $\mu$m, Merck) as packing materials. Silica gel G was used for analytical TLC.

**Plant Material.** The fresh stems of *D. crepidatum* were collected in Yunnan Province in 2008 and identified by Professor Hong Yu of Yunnan University. A voucher specimen (Zsh-8) was deposited at the State Key Laboratory of Phytochemistry and Plant Resources in West China, Kunming Institute of Botany, Chinese Academy of Science, China.

**Extraction and Isolation.** The air-dried stems of *D. crepidatum* (15 kg) were refluxed three times with 95% EtOH. After evaporation of solvent in vacuo, the residue was suspended in $H_2O$ and adjusted pH to 2 with hydrochloric acid. Then partitioned with chloroform and non-alkaloid fraction (1000 g) were obtained. Then the non-alkaloid fraction was subjected to column chromatography on silica gel (200–300 mesh, 6 kg) and eluted with petroleum ether/acetone (6:4→0:10) to afford 7 fractions. Fraction B (120g) was applied repeatedly to column chromatography over silica gel (petroleum ether/acetone, 10:1 to 1:1) and got **13** (267 mg), **14** (259 mg) and **16** (1.1 g), then residue were purified with Sephadex LH-20 ($CHCl_3$-MeOH, 1:1) to afford compounds **1** (29 mg), **4** (1.73 g), **9** (9 mg) and **10** (11 mg). Fraction C (62.7 g) was chromatographed on silica gel (petroleum ether-acetone, 5:1 to 1:1) to afford 7 fractions. Each fraction was subjected repeatedly to column chromatography over silica gel and then passed over Sephadex LH-20 (MeOH) respectively to afford compounds **5** (43 mg), **7** (11 mg) and **12** (17 mg). Fraction D was treated the same as Fraction C to afford compounds **6** (28 mg), **8** (14 mg) and **11** (300 mg). Fraction E was subjected to column chromatography over silica gel and got **15** (400 mg) and then applied to ODS column (MeOH-$H_2O$, 3:7→5:5) and Sephadex LH-20 column (MeOH) to yield compounds **2** (5 mg) and **3** (4 mg).

**Crepidatuol A (1):** yellow oil, $[\alpha]^{16.8}_{D}$ – 11.89 (*c* 0.15, $CHCl_3$), UV ($CHCl_3$): $\lambda_{max}$ (log $\varepsilon$) 282.5 (3.9) nm; IR (KBr): $\nu_{max}$ 3431, 2924, 2850, 1614, 1583, 1516, 1463, 1428, 1364, 1270, 1196, 1140, 1032 cm$^{-1}$; $^1$H NMR ($CDCl_3$, 500 MHz) and $^{13}$C NMR ($CDCl_3$, 125 MHz) data: see Table 1; EIMS m/z 436 (92), 299 (28), 285 (46), 137 (100); HREIMS *m/z* 436.1882 $[M]^+$ (calcd for $C_{26}H_{28}O_6$, 436.1886).

**Crepidatuol B (2):** yellow oil, $[\alpha]^{11.9}_{D}$ + 0.24 (*c* 0.26, $CHCl_3$), UV ($CHCl_3$): $\lambda_{max}$ (log $\varepsilon$) 282.0 (3.87) nm; IR (KBr): $\nu_{max}$ 3493, 3313, 2923, 2852, 1728, 1614, 1584, 1516, 1463, 1272, 1197, 1140, 1031 cm$^{-1}$; $^1$H NMR ($CDCl_3$, 500 MHz) and $^{13}$C NMR ($CDCl_3$, 125 MHz) data: see Table 1; EIMS m/z 452 (37), 287 (63), 137 (100); HREIMS *m/z* 452.1832 $[M]^+$ (calcd for $C_{26}H_{28}O_7$, 452.1835).

**Cell Culture and Evaluation of Neurite Outgrowth-Promoting Activity.** The neurotrophic activities of the test compounds were examined according to an assay using PC12 cells as reported.[17] Briefly, PC12 cells were maintained in F12 medium supplemented with 12.5% horse serum (HS), and 2.5% fetal bovine serum (FBS), and incubated at 5% $CO_2$ and 37 °C. Test compounds were dissolved in DMSO. For the neurite outgrowth-promoting activity bioassay, PC12 cells were seeded at a density of $2 \times 10^4$ cells/mL in 48-well plate coated with poly-L-lysine. After 24 h, the medium was changed to that containing 10 $\mu$M of each test compounds plus 10 ng/mL nerve growth factor (NGF), or various concentrations of NGF (50 ng/mL for the positive control, 10 ng/mL for the negative control). The final concentration of DMSO was 0.1%, and the same concentration of DMSO was added into the negative control and blank. After 72 h incubation, the neurite outgrowth was assessed under a phasecontrast microscope. Neurite processes with a length equal to or greater than the diameter of the neuron cell body were scored as neurite bearing cells. The ratio of the neurite-bearing cells to total cells (with at least 100 cells examined/view area; 5 viewing area/well) was determined and expressed as a percentage.

## Electronic Supplementary Material

Supplementary material is available in the online version of this article at http://dx.doi.org/ 10.1007/s13659-012-0103-3 and is accessible for authorized users.

## Acknowledgments

This work was financially supported by National Natural and Science Foundations of China (No. 30800090), "Xi-Bu-Zhi-Guang" project (2009–2012) from Chinese Academy of Science and the Fund of State Key Laboratory of Phytochemistry and Plant Resources in West China (P2010-ZZ012). The authors are grateful to the members of the analytical group of the State Key Laboratory of Phytochemistry and Plant Resources in West China, Kunming Institute of Botany, for all the spectral measurements.

**Open Access** This article is distributed under the terms of the Creative Commons Attribution License which permits any use, distribution, and reproduction in any medium, provided the original author(s) and source are credited.

## Electronic supplementary material

Supplementary material, approximately 3.25 MB.

## References

[1] Jiangsu New Medicinal University (1986). Dictionary of Chinese Medicines.

[2] Chinese Pharmacopoeia Commission (2010). Chinese Pharmacopoeia.

[3] *Flora of China*; Science Press: Beijing, 1999; Vol. 19, pp 109–110.

[4] Elander M, Leander K, Rosenblom J, Ruusa E (1973). Acta Chem. Scand..

[CR5] Hu J M, Chen J J, Yu H, Zhao Y X, Zhou J (2008). J. Asian Nat. Prod. Res..

[CR6] Hu J M, Chen J J, Yu H, Zhao Y X, Zhou J (2008). Planta Med..

[CR7] Hu J M, Zhao Y X, Miao Z H, Zhou J (2009). Bull. Korean Chem. Soc..

[CR8] Hu J M, Fan W W, Dong F W, Miao Z H, Zhou J (2012). Chin. J. Chem..

[e] Wang C Z, Jia Z J, Shen X M (1997). India J. Chem..

[6] Letcher R M, Nhamo L R M (1972). J. Chem. Soc., Perkin Trans I.

[7] Ma G X, Xu G J, Xu L S, Wang Z T, Kickuchi T (1996). Acta Pharm. Sin..

[8] Li Y, Qin L H, Annie Bligh S W, Bashall A, Zhang C F, Zhang M, Wang Z T, Xu L S (2006). Bioorg. Med. Chem..

[9] Zhang G N, Zhong L Y, Annie Bligh S W, Guo Y L, Zhang C F, Zhang M, Wang Z T, Xu L S (2005). Phytochemistry.

[10] Ma G X, Wang Z T, Xu L S, Xu G J (1998). J. Chin. Pharm. Sci..

[11] Zhang X, Gao H, Wang N L, Yao X S (2006). Chin. Tradit. Herbal Drugs.

[12] Li Y, Wang Z T, Xu L S (2006). Biochem. Syst. Ecol..

[13] Gao Y J, Zhao Q C, Min P, Shi G B, Yan M (2010). Chin. J. Med. Chem..

[14] Yu Z L, Wu Y B, Chen X F, Gao L M, Zheng Z H (2003). Chin. J. Magn. Reson..

[15] Zhang X Q, Yin Z Q, Ye W C, Zhao S X (2005). Chin. Tradit. Herbal Drugs.

[16] Greene L A, Tischler A S (1976). Proc. Natl. Acad. Sci. U.S.A..

[17] Marcotullio M C, Pagiott R, Maltese F, Obara Y, Hoshino T, Nakahata N, Curini M (2006). Planta Med..

