# Supplementary material for: Chemical components of Dendrobium crepidatum and their neurite outgrowth enhancing activities
Source: Nat Prod Bioprospect. 2013 Mar 7;3(2):70–3. doi: 10.1007/s13659-012-0103-3 (PMC4131658; doi:10.1007/s13659-012-0103-3)
Supplement: Supplementary file 1 — Supplementary material, approximately 3.25 MB. [file 13659_2012_103_MOESM1_ESM.pdf]

Electronic Supplementary Material

Chemical components of *Dendrobium crepidatum* and their neurite outgrowth enhancing activities

Cheng-Bo LI,[a] Cong WANG,[a,b] Wei-Wei FAN,[a,b] Fa-Wu DONG,[a,b] Feng-Qing XU,[a,b] Qin-Li WAN,[a] Huai-Rong LUO,[a] Yu-Qing LIU,[a] Jiang-Miao HU,[a,*] and Jun ZHOU[a]

[a]State Key Laboratory of Phytochemistry and Plant Resources in West China, Kunming Institute of Botany, Chinese Academy of Sciences, Kunming 650201, China

[b]University of Chinese Academy of Sciences, Beijing 100049, China

Received 25 December 2012; Accepted 28 February 2013

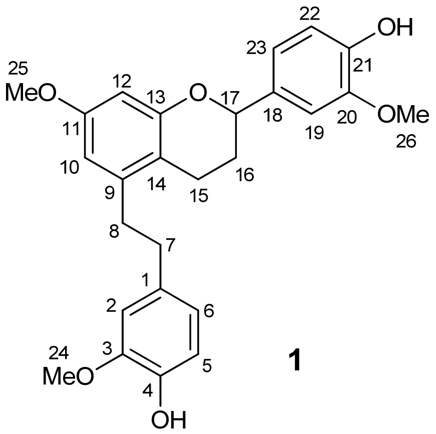

1

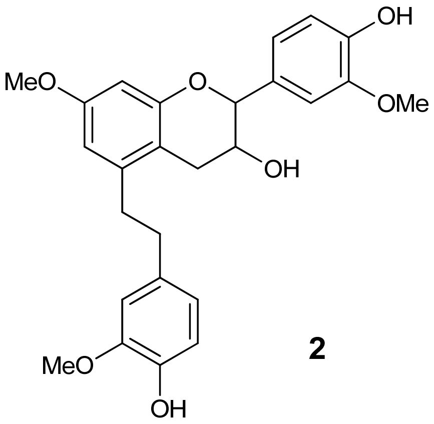

2

Structures of compounds **1** and **2**

*To whom correspondence should be addressed. E-mail: hujiangmiao@mail.kib.ac.cn

S2. EI MS spectrum of crepidatuol A (**1**)
S3. ^{1}H NMR spectrum of crepidatuol A (**1**)
S4. ^{13}C NMR spectrum of crepidatuol A (**1**)
S5. HSQC spectrum of crepidatuol A (**1**)
S6. COSY spectrum of crepidatuol A (**1**)
S7. HMBC spectrum of crepidatuol A (**1**)
S8. spectrum of crepidatuol B (**2**)
S9. ESI MS spectrum of crepidatuol B (**2**)
S10. ^{1}H NMR spectrum of crepidatuol B (**2**)
S11. ^{13}C NMR spectrum of crepidatuol B (**2**)
S12. HSQC spectrum of crepidatuol B (**2**)
S13. COSY spectrum of crepidatuol B (**2**)
S14. HMBC spectrum of crepidatuol B (**2**)
S15. spectrum of crepidatuol B (**2**)
S16. The effect of three active compounds on neurite outgrowth in PC12 cells.

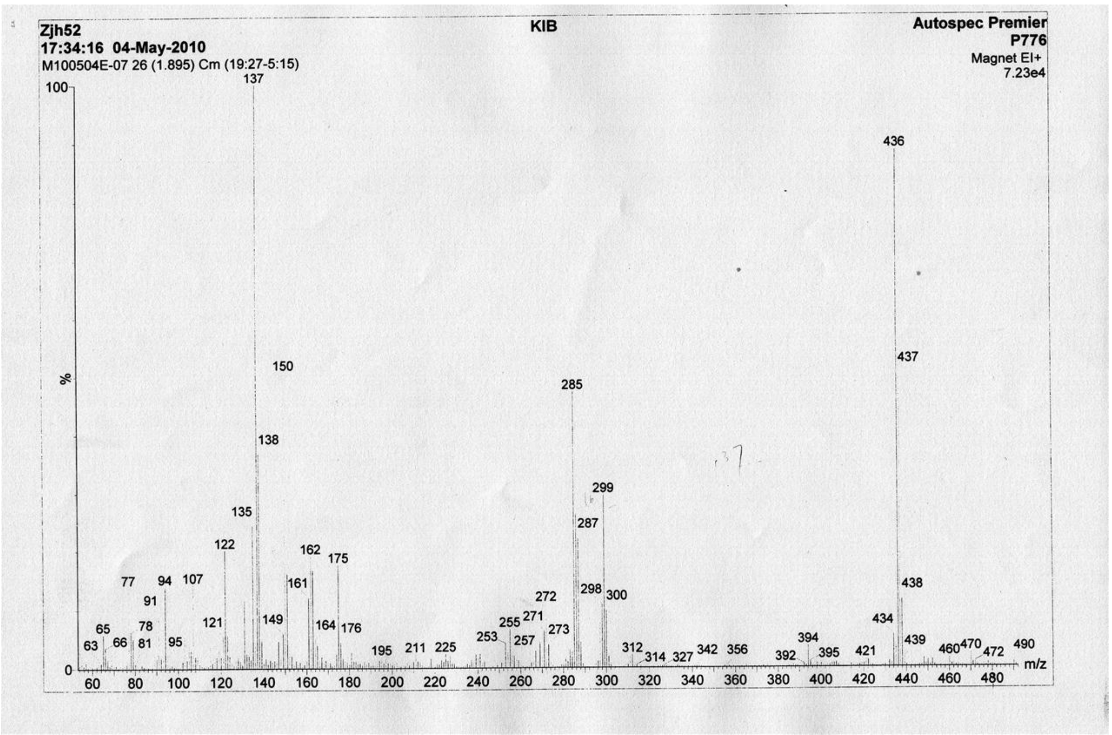

EI-MS spectrum of crepidatuol A (**1**)

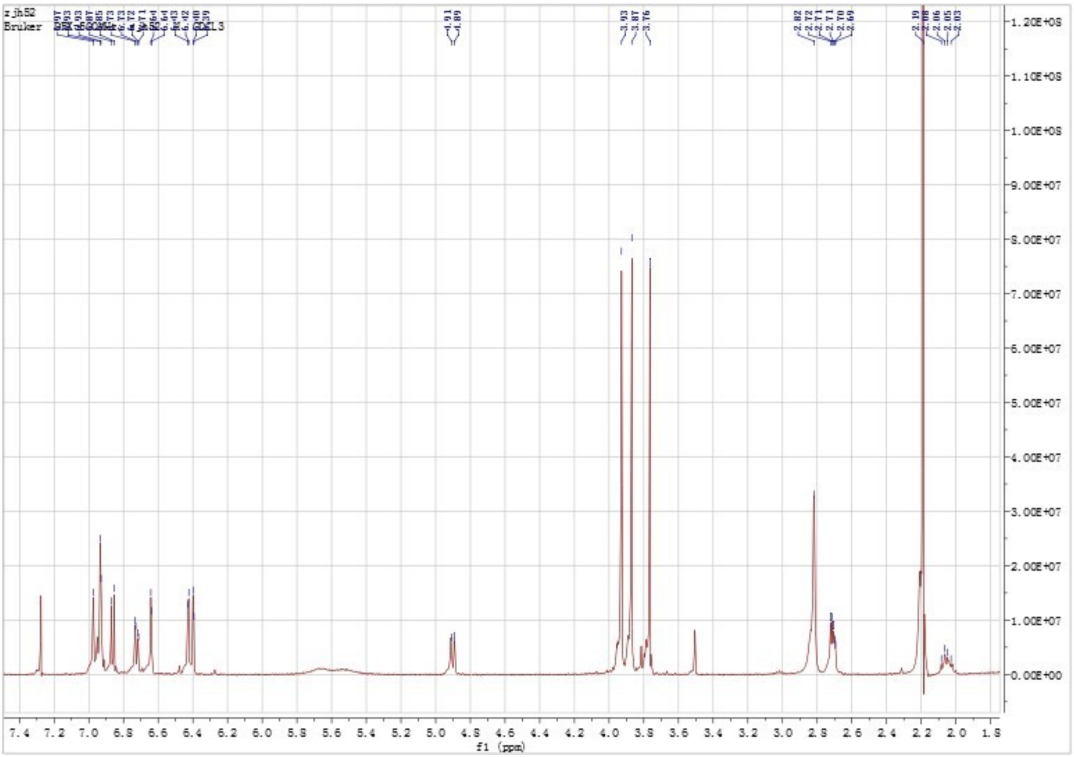

^{1}H-NMR spectrum of crepidatuol A (**1**)

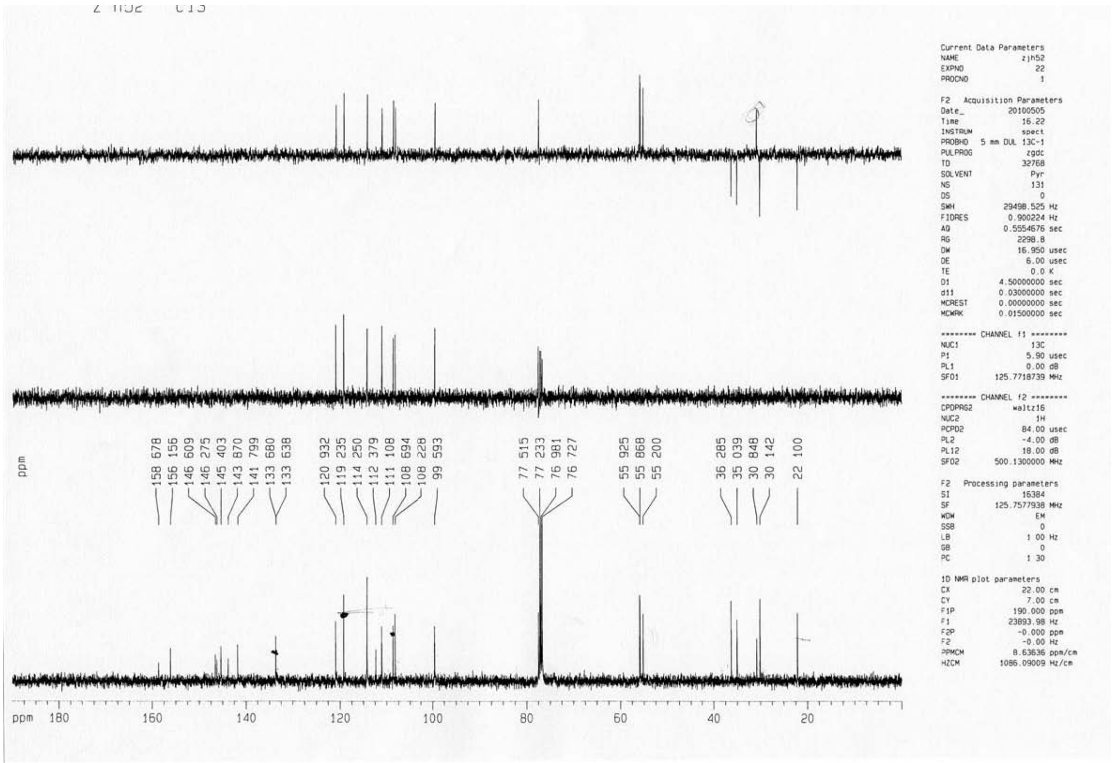

^{13}C-NMR and DEPT spectrum of crepidatuol A (**1**)

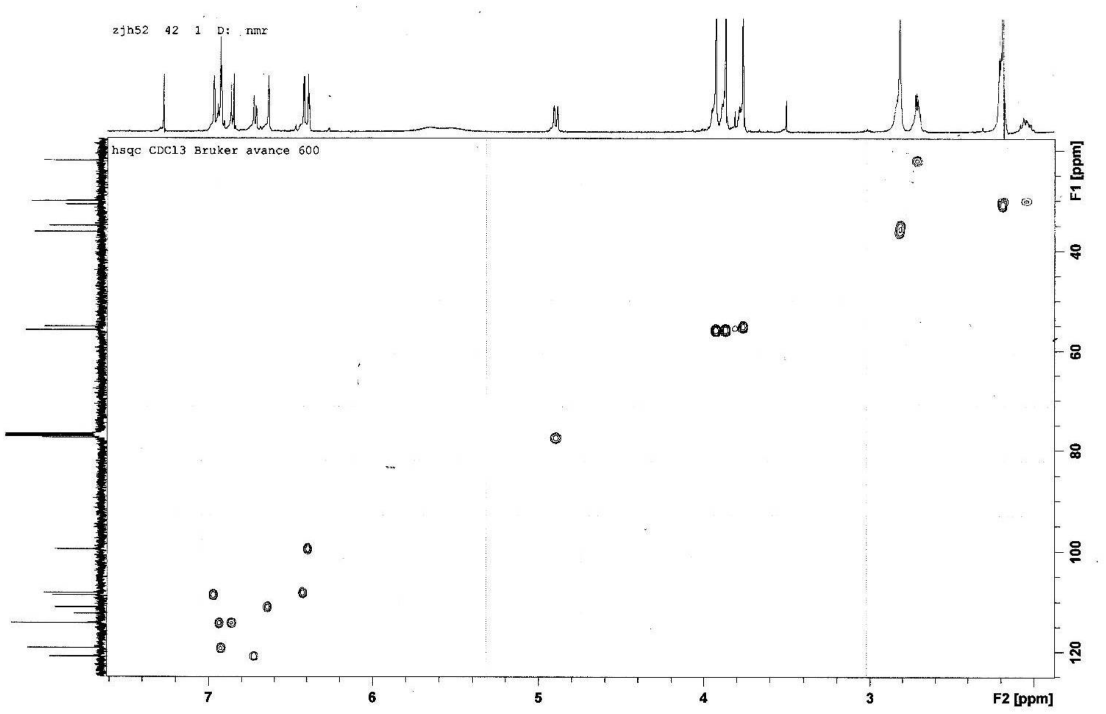

HSQC spectrum of crepidatuol A (**1**)

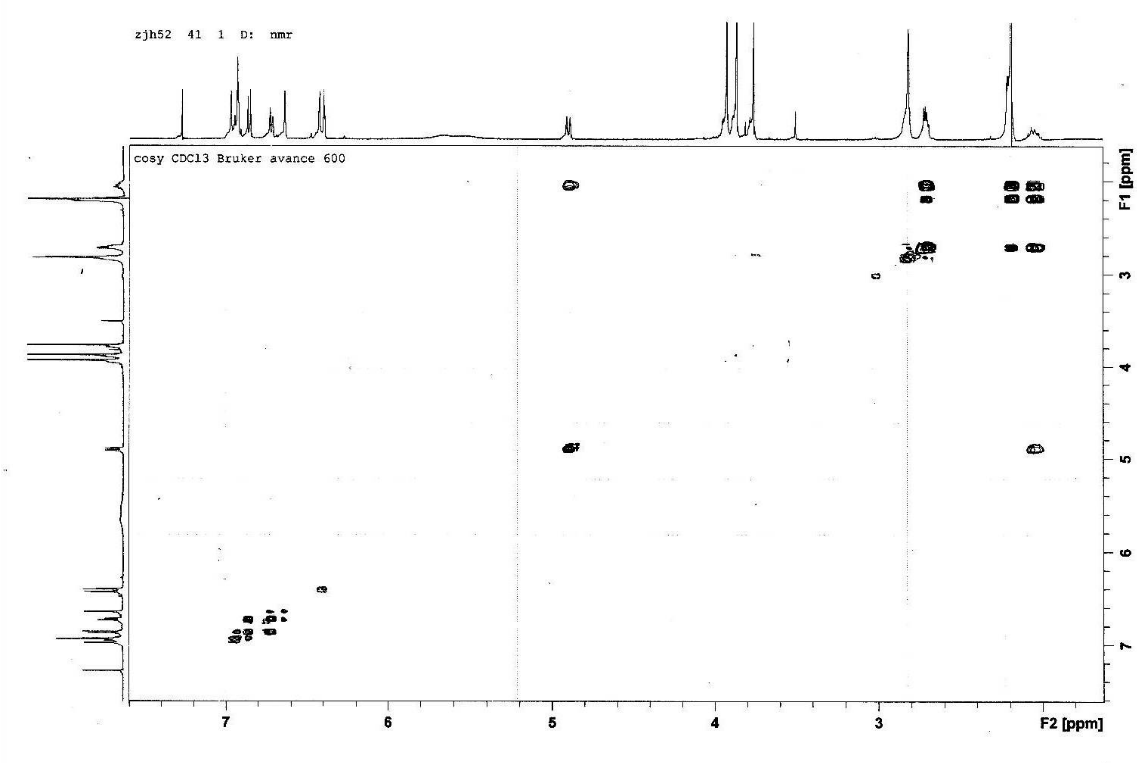

COSY spectrum of crepidatuol A (**1**)

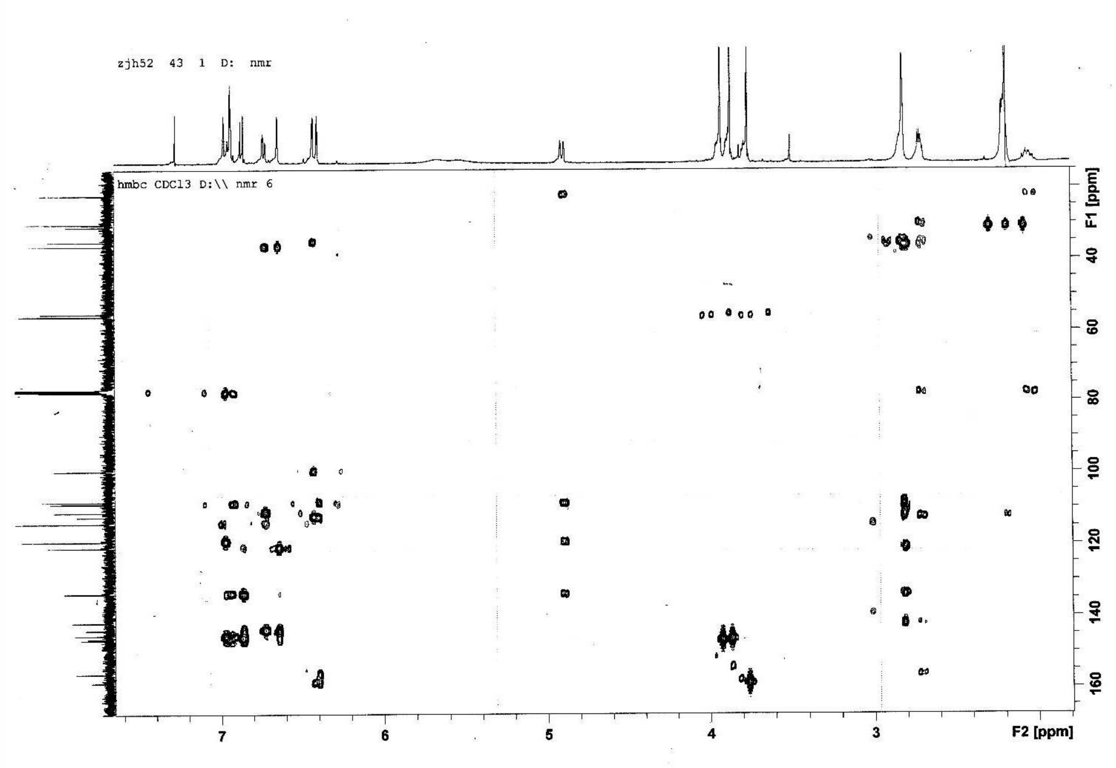

HMBC spectrum of crepidatuol A (**1**)

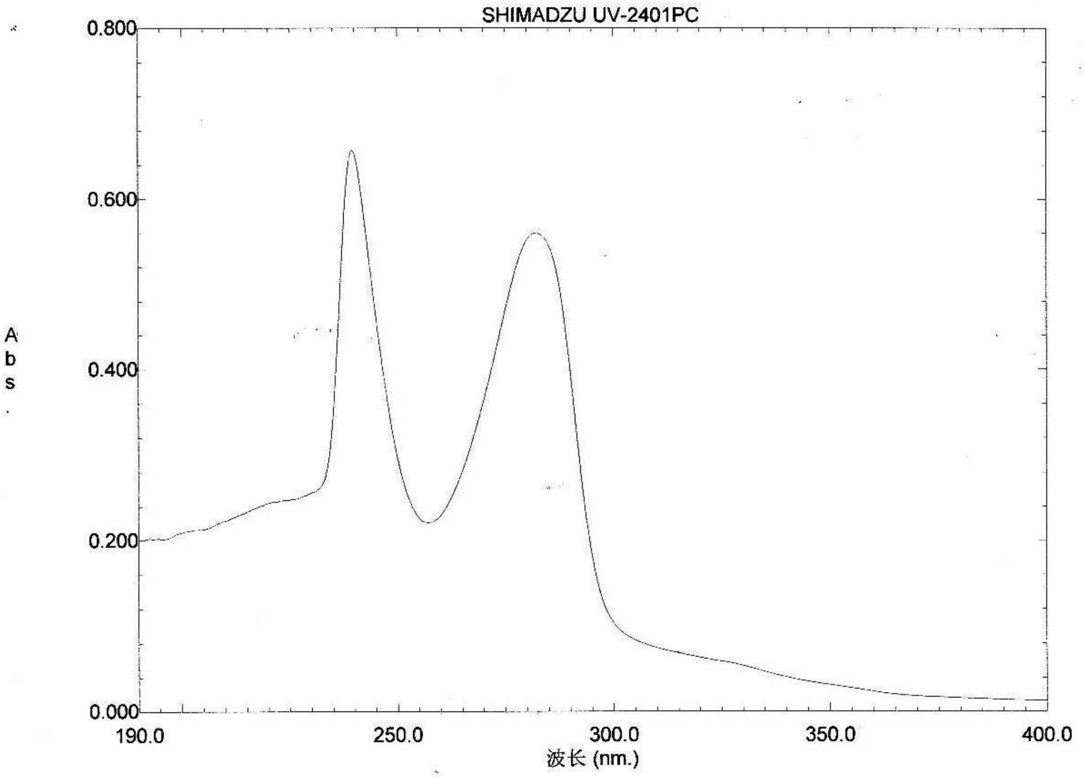

文件名: ZJH52

ZJH52 ———

样品浓度：0.0305毫克/毫升
溶剂：氯仿

创建于: 18:00 10-11-12
数据: 原始

测量模式: Abs.
扫描速度: 中速
狭缝: 5.0
采样间隔: 0.2

UV spectrum of crepidatuol A (**1**)

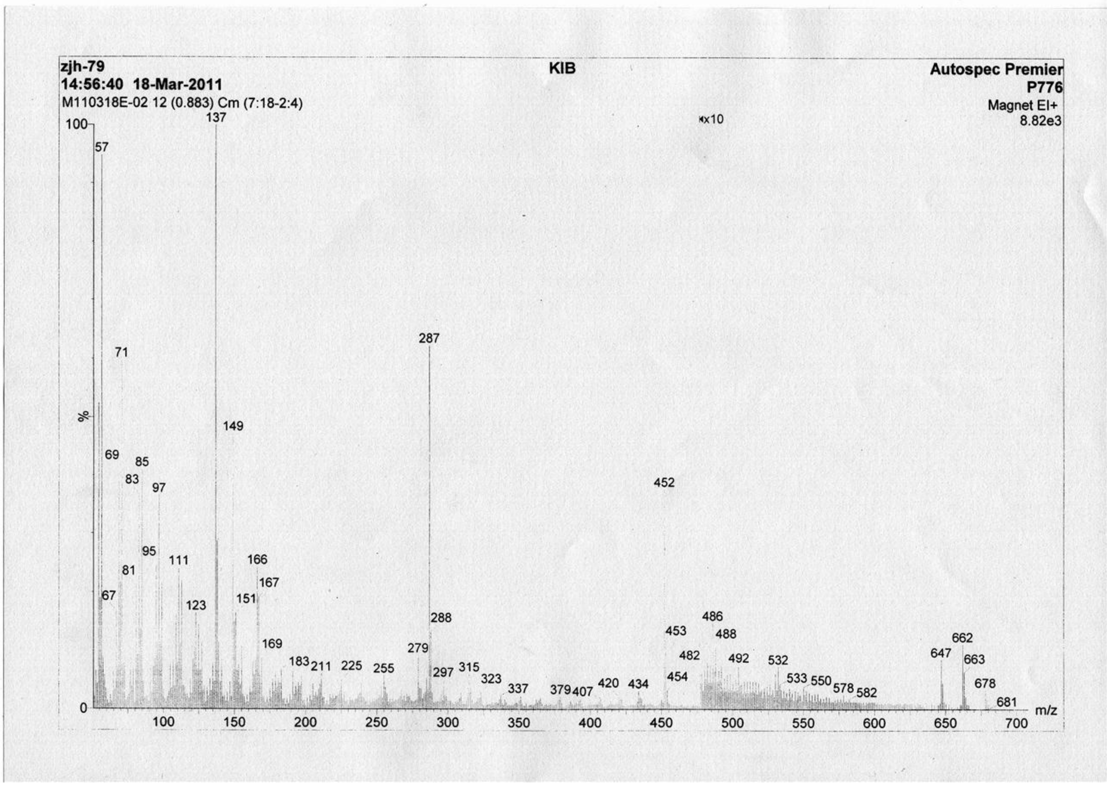

EI-MS spectrum of crepidatuol B (**2**)

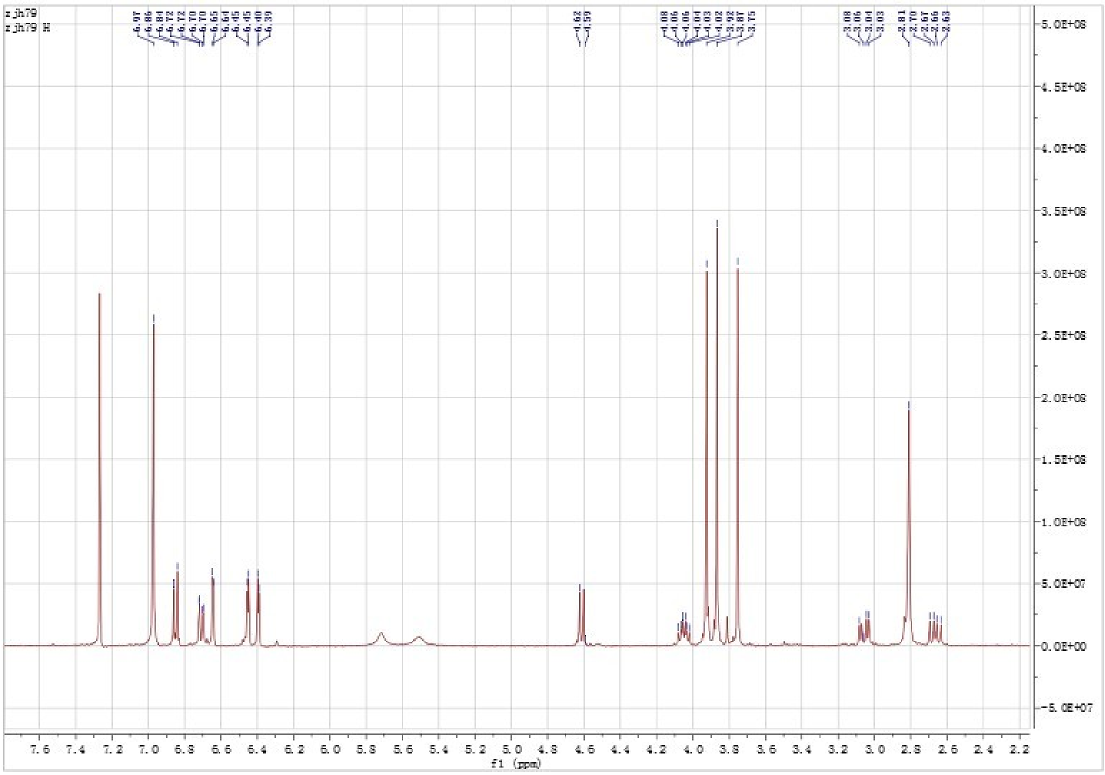

^{1}H-NMR spectrum of crepidatuol B (**2**)

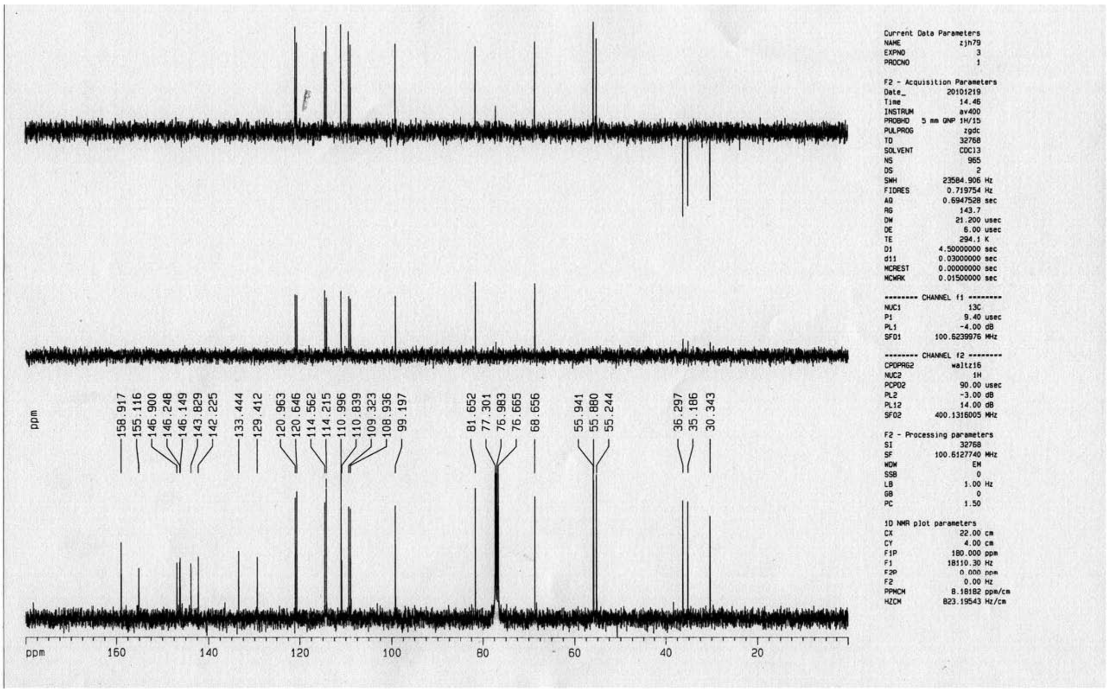

^{13}C-NMR and DEPT spectrum of crepidatuol B (**2**)

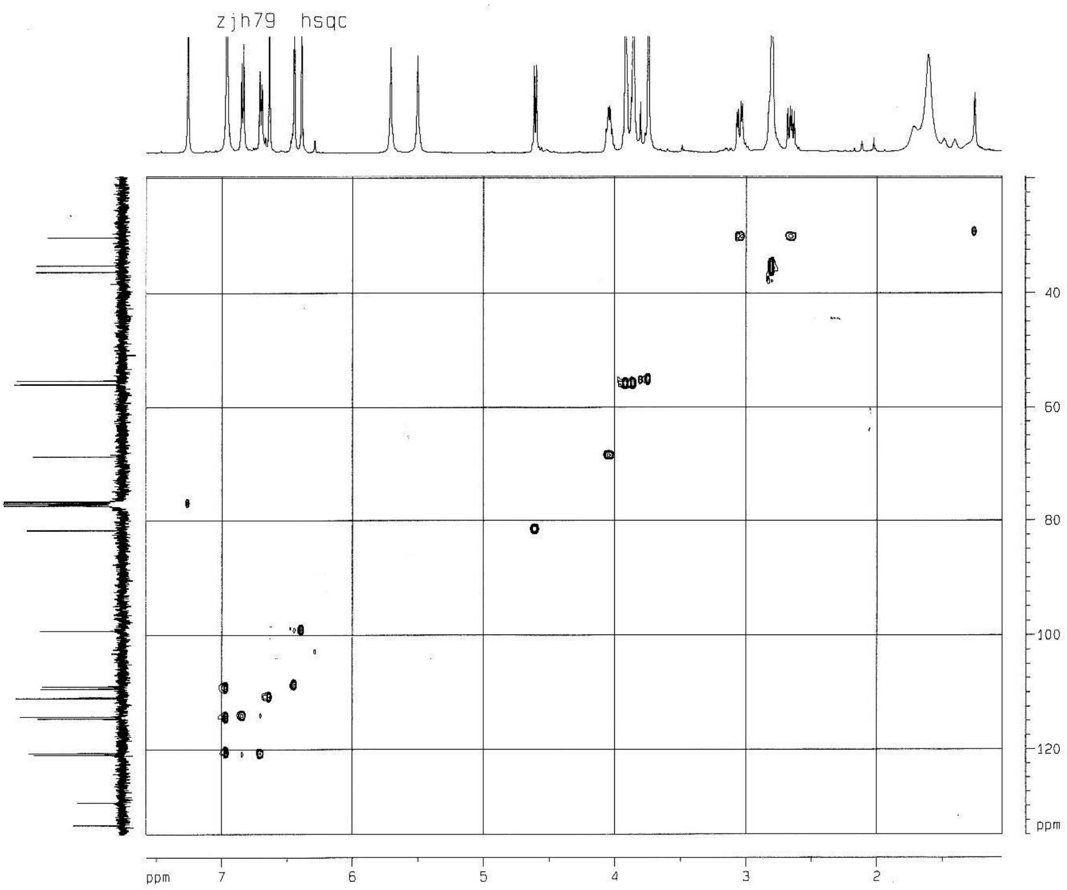

HSQC spectrum of crepidatuol B (**2**)

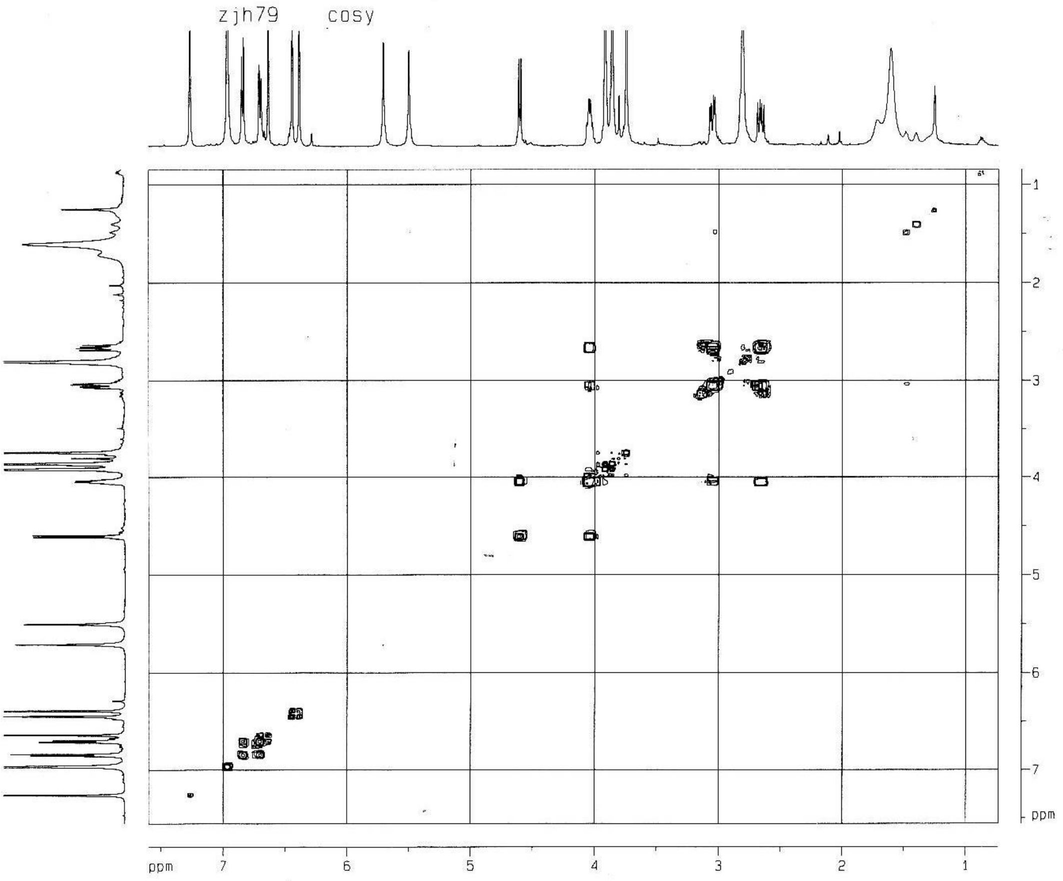

COSY spectrum of crepidatuol B (**2**)

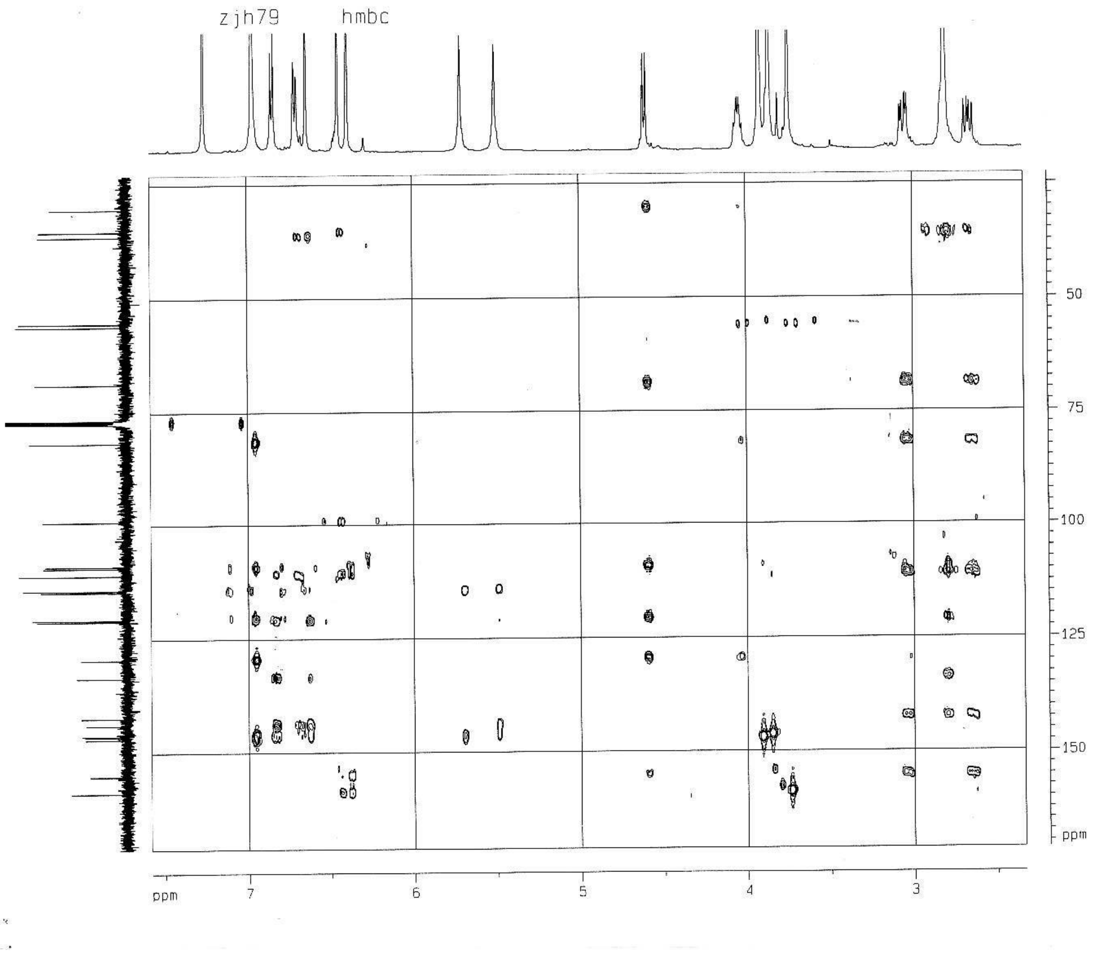

HMBC spectrum of crepidatuol B (**2**)

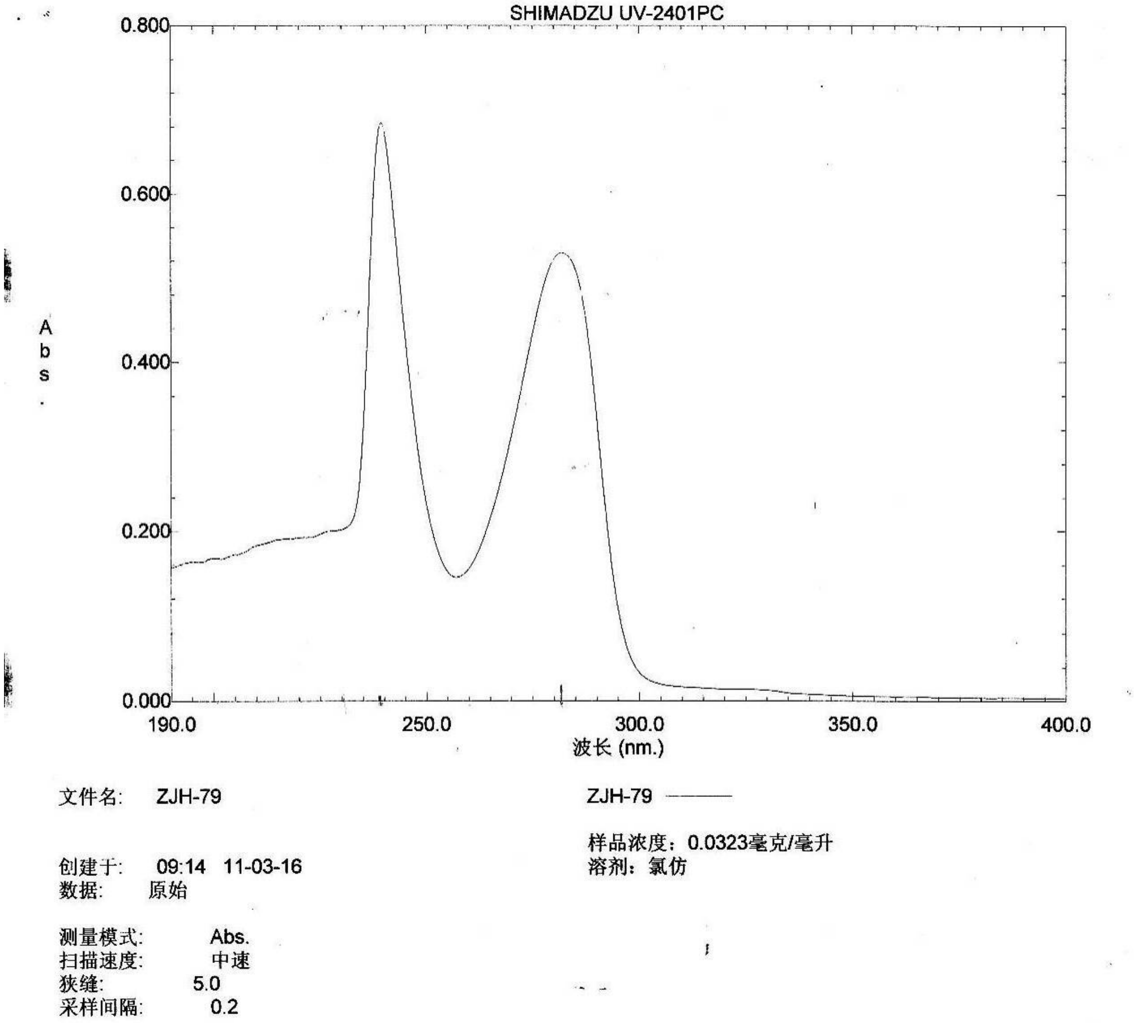

UV spectrum of crepidatuol B (**2**)

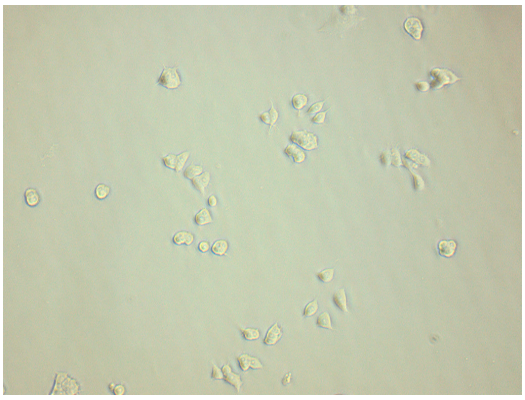

Blank

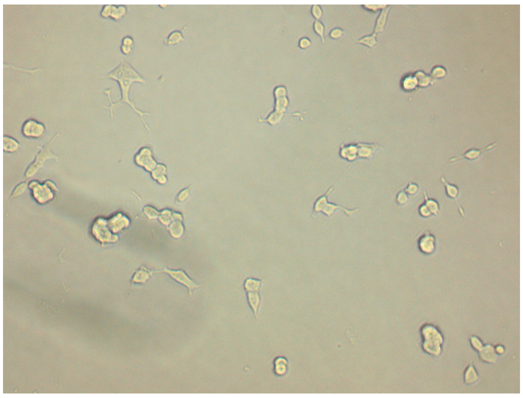

negative

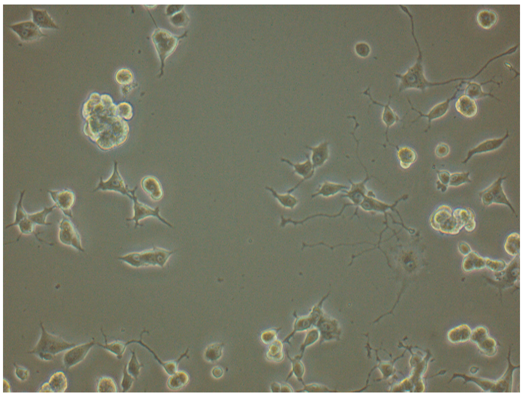

Positive

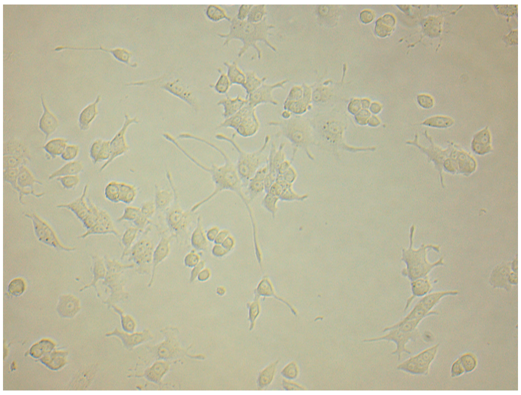

crepidatuol A;

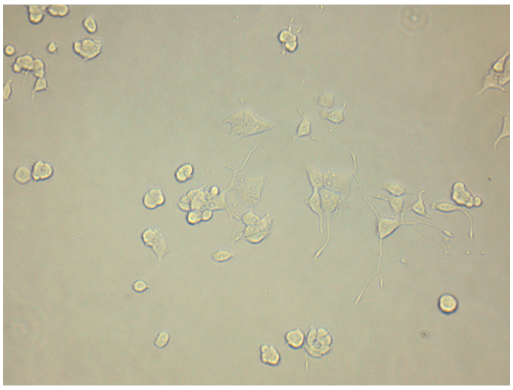

confusarin

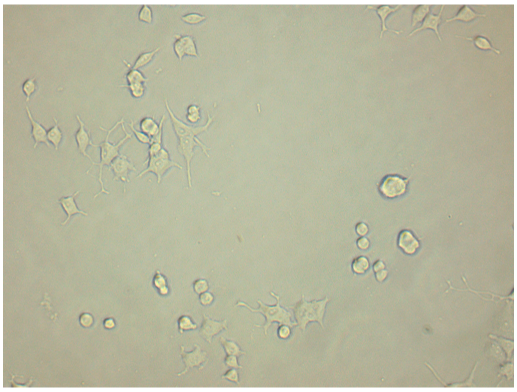

3-(2-acetoxy-5-methoxy)phenylpropanol

The effect of crepidatuol A (**1**), confusarin and 3-(2-acetoxy-5-methoxy)phenylpropanol on neurite outgrowth in PC12 cells
